# Modification of Intestinal Microbiota Dysbiosis by Low-Dose Interleukin-2 in Dermatomyositis: A *Post Hoc* Analysis From a Clinical Trial Study

**DOI:** 10.3389/fcimb.2022.757099

**Published:** 2022-03-14

**Authors:** Yunzhi Zhufeng, Jun Xu, Miao Miao, Yifan Wang, Yimin Li, Bo Huang, Yixue Guo, Jiayi Tian, Xiaolin Sun, Jing Li, Dan Lu, Zhanguo Li, Yuhui Li, Jing He

**Affiliations:** ^1^ Department of Rheumatology and Immunology, Peking University People’s Hospital, Beijing, China; ^2^ Department of Gastroenterology, Peking University People’s Hospital, Beijing, China; ^3^ Clinical Center of Immune-Mediated Digestive Diseases, Peking University People’s Hospital, Beijing, China; ^4^ Institute of Systems Biomedicine, School of Basic Medical Sciences, Peking University Health Science Center, Beijing, China; ^5^ State Key Laboratory of Natural and Biomimetic Drugs, School of Pharmaceutical Sciences, Peking University, Beijing, China; ^6^ Peking-Tsinghua Center for Life Sciences, Peking University, Beijing, China

**Keywords:** gut microbiota, idiopathic inflammatory myopathies, low-dose IL-2, Tregs, NOD mice

## Abstract

The microbiota has been observed altered in autoimmune diseases, including idiopathic inflammatory myopathies (IIMs), and associated with different treatments. Low-dose IL-2 treatment emerges as a new option for active IIMs. This study aims to explore the role of low-dose IL-2 in regulating intestinal dysbiosis involved in the IIMs. In this study, 13 patients with active IIMs were enrolled and received 1 ×10^6^ IU of IL-2 subcutaneously every other day for 12 weeks plus standard care. The clinical response and immune response were assessed. Stool samples were obtained to explore the structural and functional alterations of the fecal microbiota targeting the V3–V4 region of the 16S rRNA gene and analyze their associations with clinical and immunological characteristics. Our study demonstrated that diversity of microbiota decreased remarkably in patients with IIMs, compared to healthy controls. The inflammatory-related bacteria, such as *Prevotellaceae* increased, while some butyrate-producing bacteria, such as *Pseudobutyrivibrio, Lachnospiraceae, Roseburia*, and *Blautia*, decreased significantly. The alteration associated with disease activities in patients with IIMs. After low-dose IL-2 treatment, 92.31% (12/13) of patients achieved IMACS DOI at week 12. Proportion of Treg cells significantly increased at week 12 compared with that in baseline (15.9% [7.73, 19.4%] vs. 9.89% [6.02, 11.8%], *P* = 0.015). Interestingly, certain butyrate-producing bacteria increase significantly after IL-2 treatment, like *Lachnospiraceae, Pseudobutyrivibrio, etc.*, and are associated with a rise in L-Asparagine and L-Leucine. The effects of low-dose IL-2 on gut microbiota were more apparent in NOD mice. Together, the data presented demonstrated that low-dose IL-2 was effective in active IIMs and highlighted the potential for modifying the intestinal microbiomes of dysbiosis to treat IIMs.

## Introduction

The idiopathic inflammatory myopathies (IIMs) are a heterogeneous group of autoimmune disorders characterized by myositis and multisystemic involvement, which includes dermatomyositis (DM), antisynthetase syndrome (ASS), inclusion body myositis (IBM), and immune-mediated necrotizing myopathy (IMNM) (Mariampillai et al., 2018). The pathogenesis of IIMs remains unclear. Conventional treatment for IIMs includes glucocorticoids and immunosuppressants that are associated with substantial side effects (Oddis and Aggarwal, 2018). Several studies have shown that the low-dose interleukin-2 (IL-2) can regulate cellular immunity and promote immunological balance (Feng et al., 2019). The clinical trial suggested that low-dose IL-2 is effective in treating IIMs (Miao et al., 2021).

In recent years, several studies have demonstrated that gut microbiomes may be one of the most important contributors to the pathogenesis of rheumatic diseases. For example, microbiota alteration was observed associated with the development of rheumatoid arthritis (RA), systemic lupus erythematosus (SLE), antiphospholipid syndrome (APS), and Sjögren’s syndrome (SS) (Hevia et al., 2014; Ruff et al., 2015; Zhang et al., 2015; de Paiva et al., 2016). However, the effect of gut microbiota in IIMs has not been thoroughly studied.

Meanwhile, some studies have shown that the gut microbiota can play a role in autoimmunity by altering the abundance of microbial metabolites with immunoregulatory functions (Gaetani et al., 2020). However, there is no evidence that gut microbiota-induced metabolic alteration is associated with the treatment of IIMs. In this study, we investigated the effects of low-dose IL-2 on gut microbiota and metabolism in patients and rats. In addition, we performed a correlation analysis between gut microbiota and clinical indicators to decipher the possible roles in the pathogenesis of IIMs.

## Methods

### Subjects Enrollment

This was a *post hoc* analysis of data from a prospective cohort study of low-dose IL-2 in myositis patients[NCT04062019]. A total of 13 Chinese patients with IIMs, whose diagnoses were based on the 2017 EULAR/ACR criteria (Lundberg et al., 2017), or ASS proposed by Solomon et al. (Solomon et al., 2011), from November 2019 to June 2020, with 52 healthy controls. Low-dose interleukin-2 was injected subcutaneously at a dose of 1×10^6^ IU once every other day for 12 weeks. Fresh blood samples were used to assay blood indicators. The clinical index and laboratory parameters were evaluated before and after treatment.

### Animals

Female NOD mice aged seven weeks were purchased from the model animal research center of Nanjing University and raised in pathogen-free conditions in Peking University People’s Hospital. Low-dose IL-2 was injected subcutaneously at a dose of 30,000 IU every other day for 60 days (Li et al., 2017). After that, the spleen, the inguinal, and cervical lymph nodes were obtained for flow cytometry.

### Flow Cytometric Analysis

The splenic and lymph nodes of mice were collected and resuspended in RPMI1640 medium containing 10% fetal bovine serum (FBS; Sigma-Aldrich, St. Louis, MO). We stained the cells with a combination of fluorescence-conjugated monoclonal antibodies CD3, CD4, CD8, PD-1, CXCR5, B220^+^, CD25, Foxp3, IFN-γ, and IL-17A antibodies by intracellular staining (Qin et al., 2019). A BD FACS Aria II (BD Biosciences) instrument and Kaluza Analysis software analyzed and screened the stained samples.

### Fecal Sample Collection and DNA Extraction

Approximately 2 g of fresh fecal sample was collected from each subject and stored at -80°C. According to the instructions, DNA was extracted from homogenized feces using the QIAamp DNA Stool Mini Kit. The integrity and size were verified by electrophoresis on a 1.2% agarose gel.

### Amplicon Library Construction and Sequencing

Amplicon libraries were constructed with Illumina sequencing-compatible and barcode-indexed bacterial PCR primers 357F (5’-ACTCCTACGGRAGGCAGCAG-3’)/806R (5’-GGACTACHVGGGTWTCTAAT-3’), which target the V3-V4 regions of the 16S rRNA gene. All PCR reactions were performed with ABI 9700 using the manufacturer’s protocol. Thermocycling conditions were set at 94°C for 2 min and then 94°C for 30 s, 56°C for 30 s, 72°C for 30 s for 25 cycles, followed by a final extension at 72°C for 5 min.

### Bioinformatics

Vsearch was applied to filter the low-quality sequences, and amplicons were generated using Sliva_16s_v123.fa (Rognes et al., 2016). To determine differences of fecal microbiota between the IIMs patients and health groups, we analyzed the α diversity and β diversity by QIIME. Chao1, richness, natural logarithm value of Shannon index, and equitability indices were assessed to determine α diversity. Principal coordinates analysis (CPCoA) with Adonis test was used to calculate the bacterial β diversity. Bacterial signaling pathways were analyzed using PICRUSt2 (Douglas et al., 2020). Correlation between bacterial and IIMs complications was assessed by the MaAsLin2 method (Sun et al., 2021). Spearman’s test evaluated correlation between bacterial and lymphocyte or metabolic indexes.

### Measurement of Plasma Amino Acids

#### Sample Pretreatment

Study plasma samples, preserved at -80°C low temperature, were thawed at 4°C. Each sample (20μL) was added followed by internal standard solution (10μL 0.1mM L-Norvaline) and acetonitrile (70μL). The mixture was vortexed for 20s, and then, centrifuged for 10min at 15000g. Take 10μL of the supernatant, and derivate according to the Waters AccQ Tag method. Then, 70μL Buffer was added, and the mixture was vortexed for 10s. Next, add 20μL derivate, vortex immediately and stand for 1min, then stand for 10min at 55°C and immediately vortex again. The final mixtures were stored at 4°C before detection.

#### LC-MS/MS Analysis

The amino acid components were identified and quantified using Eksigentultral liquid chromatography 100 couples with AB 5600 Triple TOF system (AB SCIEX), and separated using 2.1×100mm XBridge Peptide BEH C18 column (waters) with a 4×2.0mm guard column (phenomenex). The separation of amino acids was achieved under a column temperature of 50°C using ammonium formate (10mM), formic acid (0.1%, v/v) and water (99.9%, v/v) as mobile phase A, formic acid (1.6%, v/v) and acetonitrile (98.4%, v/v) as mobile phase B. The step gradient was as follows: 0.01min, 5% (v/v) B; 0.01-15min, 5% to 40% (v/v) B; 15-17min, 40% to 100% (v/v) B; 17-20min, 100% (v/v) B; 20-20.5min, 100% to 5% (v/v) B; 20.5-25min, 5% (v/v) B. The injection volume was 5μL, and the total run-time was 25 min at a flow rate of 0.4 mL/min. The instrument, under the positive model, was set as follows: curtain gas, ion source gas 1 and ion source gas 2 at 30, 50, 50psi, respectively; source temperature at 550°C and ion spray voltage floating at 5500V. In auto MS/MS acquisition, the m/z range for TOF MS scan and production of ion scan were 200-700Da and 50-700Da, respectively. The collision energy of the product ion scan was set at 30 ± 15V and the declustering potential was set at 80V.

#### Data Processing

The Peak View 1.2 was used to identify amino acids and Multi Quant 2.1 was used to quantify amino acids based on the m/z value and sample retention time.

### Statistical Analysis

The Mann-Whitney U test and Wilcoxon signed-rank test were used for continuous variables and the *Chi-squared* test for categorical variables. All trials of significance performed were two-sided, with *P* ≤ 0.05 or corrected *P* (FDR) ≤ 0.05 considered statistically significant.

## Results

### Gut Microbial Dysbiosis Is Detectable in IIMs Compared With Healthy Controls

Seventy-eight fecal samples were collected from 13 IIM patients (before and after Low-dose IL2 treatment) and 52 healthy controls. 4,882,310 high-quality sequences were kept for further bioinformatic analysis after filtering low-quality sequences and the redundant sequences. 4248 amplicons were generated and were subjected to denoise and filtering chimeras with reference of Sliva_16s_v123.fa (Edgar, 2016). 2232 amplicon sequence variants (ASVs) have been identified for the generation of the ASV table. As a result, 3 ASVs were presented in all samples (0.1%), and 37 (1.7%) and 354 ASVs (15.9%) were found in 90% and 50% samples.

**Figure 1:**
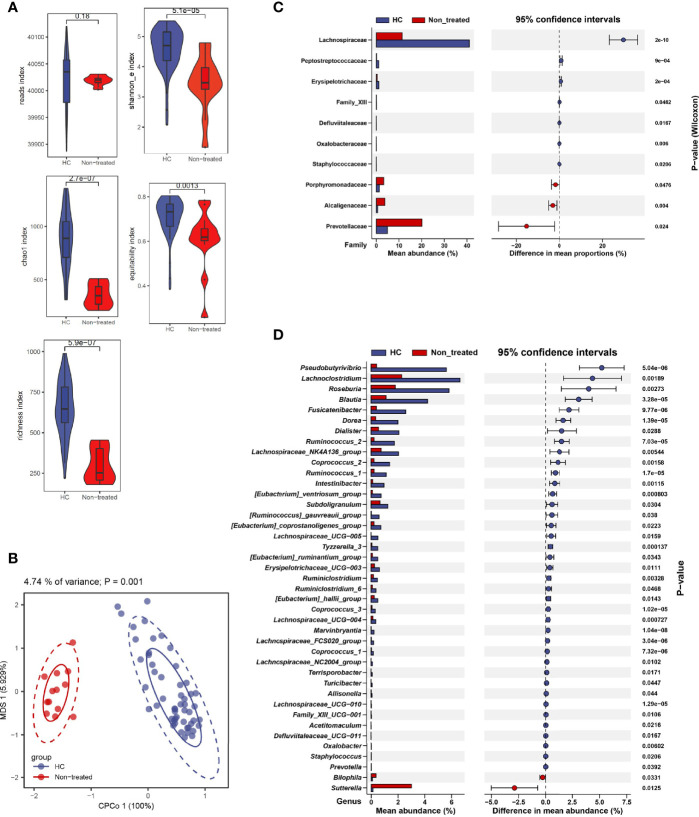
Alteration of fecal microbiota in patients with idiopathic inflammatory myopathies. **(A)** α-diversity is measured by reads, chao1, richness, Shannon_e, and equitability indexes. **(B)** β-diversity measured using the Adonis test by conditioned constrained principal coordinate analysis (CPCoA). **(C)** Significant differences of bacteria between the two groups at the family level. **(D)** Significant differences of bacteria between the two groups at the genus level. Data visualization was performed using the R software (version 4.0.3) with the *ggplot2* package.

Indexes including chao1 (*P* = 2.7e-07), richness (*P* = 5.9e-07), Shannon_e (based on natural logarithm, *P* = 5.1e-05), and equitability (*P* = 0.0013) were significantly changed in IIMs compared with the healthy controls from the perspective of alpha diversity **(** **Figure 1A** **)**. Result from beta diversity with constrained principal coordinates analysis (CPCoA) also showed a dramatic difference in the bacterial community between IIMs patients and healthy controls (**Figure 1B**, *Adonis P* = 0.001).

We identified gut microbiota composition with marked difference. Data showed that, at the family level, Porphyromonadaceae (*P* = 0.0476), Alcaligenaceae (*P* = 0.004), and Prevotellaceae (*P* = 0.024) family were increased in IIMs patients compared with healthy controls **(** **Figure 1C** **)**. At the genus level, *Pseudobutyrivibrio* (*P* = 5.04e-06), *Lachnoclostridium* (*P* = 0.00189), *Roseburia* (*P* = 0.00273), *Blautia* (*P* = 3.28e-05), etc. were significantly decreased in IIMs patients, while *Bilophila* (*P* = 0.0331) and *Sutterella* (*P* = 0.0125) were increased in IIMs group **(** **Figure 1D** **)**.

### Correlation Between Bacterial Community and Immune Parameters

**Figure 2:**
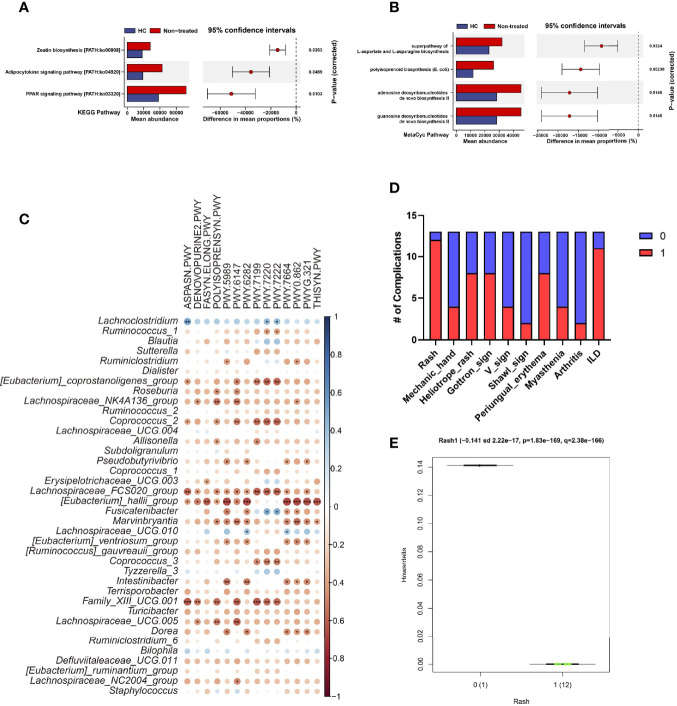
Characteristic of the composition and function of the gut microbiota in IIMs patients. The online PICRUSt2 was performed to analyze the bacteria-associated signaling pathways. Presenting signaling pathways were enriched depending on the KEGG **(A)** and MetaCyc pathway databases **(B). (C)** Correlations between signaling pathways and bacteria. Data visualization was performed using the R software (version 4.0.3) and the *corrplot* package (^*^ *P*<0.05; ^**^ *P*<0.01; and ^***^ *P*<0.001). **(D)** Percentage of IIMs complications. **(E)** The *Howardella* richness was significantly correlated with IIM involved Rash. The multivariate analysis was performed to test the correlation between clinical parameters and bacterial microbiome with the online MaAsLin2 (http://huttenhower.sph.harvard.edu/galaxy/).

To investigate bacterial signaling pathways, PICRUSt2 was used for the analysis of KEGG orthology (KO), enzyme (EC), KEGG pathway, and MetaCyc pathway, respectively. Data showed Glycine reductase, Aspartyl-tRNA synthetase were reduced, and threonine synthase (thrC) and 23S rRNA pseudouridine 2605 synthase (rluB) were increased in IIMs patients at both EC and KO levels, respectively **(** **Supplementary Figures 1A, B** **)**. Based on KEGG pathway analysis, Zeatin biosynthesis, Adipocytokine, and PPAR signaling were enriched in IIMs patients **(** **Figure 2A** **)**; and L-aspartate and L-asparagine, polyisoprenoid, adenosine, and guanosine deoxyribonucleotides *de novo* biosynthesis were enriched in MetaCyc pathway analysis **(** **Figure 2B** **)**. Further investigation revealed that the enriched bacteria in healthy controls were negatively correlated with the pathways involved in IIMs, including Lachnospiraceae_FCS020_group, *[Eubacterium]_hallii_group*, *Family_XIII_UCG.001*, and *Dorea* **(** **Figure 2C** **)**.

Next we analyzed the correlation between bacterial contents and clinical indices for IIMs with the MaAsLin2 method (Sun et al., 2021) **(** **Figure 2D** **)**. Results showed that the genus *Howardella* in IIMs patients positively correlated with rash (*P* = 1.83e-169, *Q* = 2.38e-166) **(** **Figure 2E** **)**. There was no evidence to show the relationship between bacterial genera and other clinical features.

We also investigated the alteration of immune and metabolic status in IIMs patients before and after low-dose IL-2 treatment (for 12 weeks in 13 individuals). Fifty immune-associated parameters and twenty-six metabolic indexes were collected for comparative analysis.

**Figure 3:**
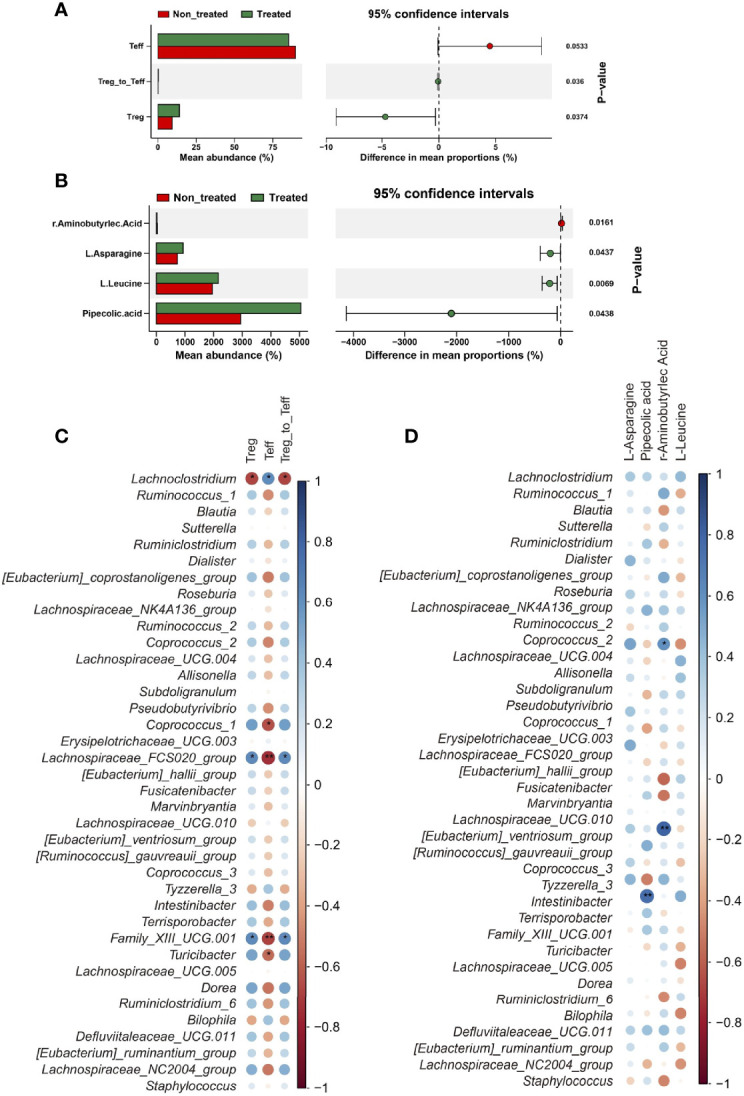
IL-2 treatment recovered the immune balance and altered the metabolism in myositis patients. **(A)** Significant differences of immune cells between the two groups. **(B)** Significant differences in amino acid metabolism between the two groups. Data visualization was performed using the R software (version 4.0.3) with the ggplot2 software. **(C)** Correlations between immune parameters and bacteria genera. **(D)** Correlations between metabolic parameters and bacteria genera. Data visualization was performed using the R software (version 4.0.3) and the *corrplot* package (^*^ *P*<0.05; ^**^ *P*<0.01).

Data showed Tregs, Teff cells, and the ratio of Treg to Teff were significantly different between before and after IL-2 treatment groups (non-treated and treated groups) **(** **Figure 3A** and **Supplementary Table 1** **)**. Consist with our previous studies, the proportion of Treg cells was significantly increased after low-dose IL-2 therapy (9.89% [6.02, 11.8%] vs. 15.9% [7.73, 19.4%], *P* = 0.015) **(** **Table 1** **).** Meanwhile, serum γ-aminobutyric acid (GABA) decreased in IIMs patients after IL-2 treatment, with an increase of L-Asparagine, L-Leucine, and Pipecolic acid. However, there were few significant changes in the metabolic contents in the fecal specimens after low-dose IL-2 therapy **(** **Figure 3B** and **Supplementary Table 2** **)**.

**Table 1:** Response of IIMs patients to low-dose IL-2 treatment.

Characteristics	Non-treated	Treated	*P* value
IMACS DOI, n (%)	–	12 (92.31)	–
CSM			
PhGA-VAS, median (range)	6 (5, 7)	3 (2, 4)	0.001***
PGA-VAS, median (range)	6(5, 6.5)	3 (2, 4)	0.001***
MMT-8 (0-150), median (range)	146 (136, 150)	150 (141, 150)	0.018*
HAQ-DI (1-3), median (range)	0.3 (0, 1)	0.3 (0, 1)	0.18
Extra muscular disease, VAS (0-10)	5 (4, 6)	2 (1.13, 3.75)	0.002**
ALT, median (range)	22 (13, 36)	15.5 (10.75, 22)	0.037*
AST, median (range)	25 (20.5, 44)	15.5 (12.5, 28.75)	0.013*
LDH, median (range)	246 (207, 346)	225 (179, 327.25)	0.158
CK, median (range)	218 (39.5, 271)	50.5 (26, 179.5)	0.071
CDASI-a (0-100), median (range)	12 (5, 14.5)	3 (0, 5.5)	0.001**
CDASI-d (0-32), median (range)	1 (0, 2)	0 (0, 1.5)	0.034*
Fatigue-VAS (0-10), median (range)	4 (2, 6.75)	3 (2, 4.75)	0.018*
Rash, n (%)	12 (92.31)	6 (46.15)	0.011*
Mechanic hands, n (%)	4 (30.77)	2 (15.38)	0.348
Heliotrope rash, n (%)	8 (61.54)	3 (23.08)	0.047*
Gottron’s sign/papules, n (%)	8 (61.54)	3 (23.08)	0.047*
V sign, n (%)	4 (30.77)	1 (7.69)	0.135
Shawl sign, n (%)	2 (15.38)	1 (7.69)	0.539
Periungual erythema, n (%)	8 (61.54)	0 (0)	0.001***
Arthritis, n(%)	2 (15.38)	1 (7.69)	0.539
ILD, n (%)	11 (84.62)	11 (84.62)	>0.99
Malignancy, n (%)	0 (0)	0 (0)	>0.99
ESR, median (range)	14 (10.5, 29.5)	17.5 (8, 29)	0.875
CRP, median (range)	2.15 (0.5, 20.62)	1.45 (0.13, 10.85)	0.345
C3, median (range)	0.86 (0.77, 1.13)	1.05 (0.91, 1.44)	0.009**
C4, median (range)	0.19 (0.16, 0.26)	0.25 (0.19, 0.31)	0.008**
Treg, (% in CD4 T)	9.89 (6.02, 11.8)	15.9 (7.73, 19.4)	0.015*
Teff, (% in CD4 T)	89.6 (86.95, 93.45)	83.5 (80.18, 92.08)	0.028*
Treg/Teff	0.11 (0.06, 0.14)	0.11 (0.06, 0.14)	0.015*

Data are presented as median (IQR), mean ± Std or n (%). IIMs, idiopathic inflammatory myopathies; IMACS DOI, International Myositis Assessment and Clinical Studies (IMACS) definition of improvement (DOI); PhGA, physician’s global assessment of disease; VAS, visual analog scale; PGA, patient’s global assessment of disease; MMT-8, Manual Muscle Test-8; HAQ-DI, the health assessment questionnaire disability index; ALT, alanine transaminase; AST, aspartate transaminase; LDH, lactate dehydrogenase; CK, creatinine kinase; CDASI-a, Cutaneous Dermatomyositis Disease Area and Severity Index Activity Score; CDASI-d, Cutaneous Dermatomyositis Disease Area and Severity Index Damage Score; MDAAT, Myositis disease activity assessment tool; ILD, interstitial lung disease; ESR, erythrocyte sedimentation rate; CRP, C-reactive protein; C3, complement 3; C4, complement 4; Treg, regulatory T cell; Teff, effector T cell (*P<0.05; **P<0.01 and ***P<0.001).

### Efficacy and Modification of Microbial Dysbiosis by Low-Dose IL-2 Therapy

After three months of treatment with low-dose IL-2, 12 out of 13 (92.31%) patients were responders and reached the IMACS DOI **(** **Table 1** **)**. MMT-8 was increased from 146 (136, 150) at baseline to 150 (141, 150) (*P* = 0.018) after IL-2 treatment. Details of improvements in other CSMs, including PhGA, PGA, and HAQ-DI, muscle enzymes, and extra muscular activity, were summarized in **Table 1**. The extra muscular global, primarily driven by skin rash in our study, showed a median of 50% improvement in all patients. The cutaneous dermatomyositis disease area and severity index activity score (CDASI-a) was decreased significantly from 12 (5, 14.5) to 3 (0, 5.5) after IL-2 administration (*P*=0.001). The improvement of skin rash included mechanic’s hands (in two of the four patients with this manifestation), heliotrope rash (5 of 8 patients), Gottron’s sign/papules (5 of 8 patients), V sign (in 3 of the 4 patients with this manifestation), Shawl sign (1 of 2 patients), and periungual erythema (in all of 8 patients with this manifestation) **(** **Table 1** **)**.

We further evaluated the correlation between bacterial genera and immune/metabolic parameters. In these markedly changed bacterial genera, four of Clostridiales order, *Coprococcus_1*, *Family_XIII_UCG.001*, and Lachnospiraceae_FCS020_group, were positively correlated with Treg cells, and Treg/Teff **(** **Figure 3C** **)**. Notably, only a few correlations were found between the bacterial genera and metabolic indexes **(** **Figure 3D** **)**.

### Bacterial Swing/Oscillation in IIMs Patients With Low Dose IL-2 Treatment

**Figure 4:**
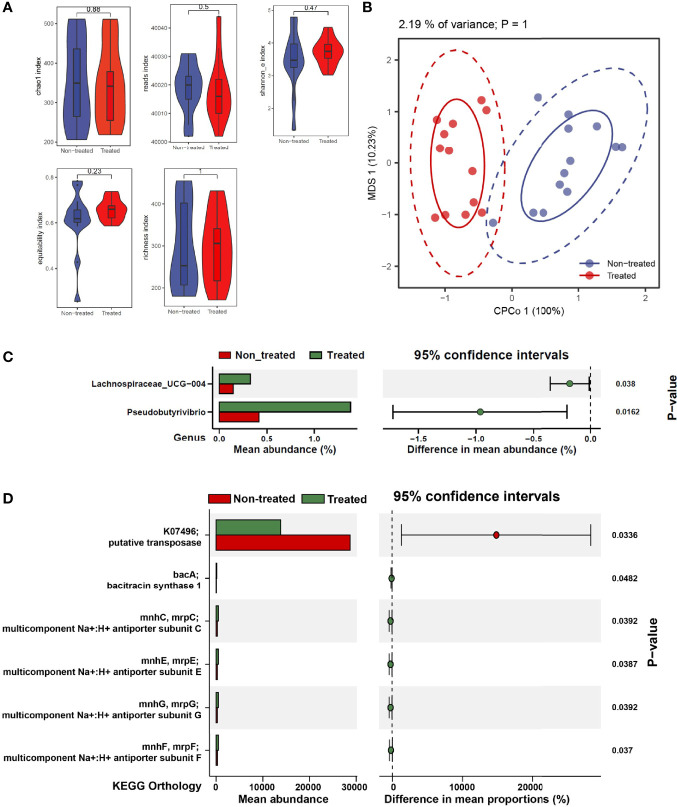
The effects of low-dose IL-2 on regulating gut microbiota composition and function in IIMs patients. **(A)** The α-diversity is measured by reads, chao1, richness, Shannon_e, and equitability indexes. **(B)** The β-diversity measured using the Adonis test by conditioned constrained principal coordinate analysis (CPCoA). **(C)** Significant differences of bacteria between the two groups at the family level. **(D)** The online PICRUSt2 was performed to analyze the bacteria-associated signaling pathways. Presenting signaling pathways were enriched depending on the KEGG database. Data visualization was performed using the R software (version 4.0.3) with the *ggplot2* package.

To investigate the role of IL-2 treatment on the bacterial community, we tested the longitudinal changes of bacterial contents in IIMs patients. There was no significant alteration in bacterial alpha diversity and beta diversity after IL-2 treatment **(** **Figures 4A, B** **)**. A comparative analysis was also performed in the bacterial abundance at the family and genus levels before and after low-dose IL-2 treatment. The genus of Lachnospiraceae_UCH-004 and *Pseudobutyrivibrio* significantly increased in IIMs patients after IL-2 treatment **(** **Figure 4C** **)**. No widely modified bacterial families were found after IL-2 treatment, Additional bacterial signaling showed that the suspected transposase could not be detected after administration of IL-2, with enrichment of bacitracin synthase 1 and several subunits of the Na:H multi-component carrier **(** **Figure 4D** **)**.

### Evaluating the Role of IL-2 Treatment on Fecal Microbiota With the NOD Mouse Model

The microbial community of patients is always influenced by various environmental and clinical factors, such as demography, diet, complications, *etc*. Therefore, we chose the NOD mice, a model for autoimmune diseases (Aoki et al., 2005; Kim et al., 2020), to better evaluate the influence of IL-2 treatment on gut microbiota. The Shannon and equitability indexes in each sample revealed that alpha diversity were changed after low-dose IL-2 therapy **(** **Supplementary Figure 2A** **)**. Beta diversity based on the CPCoA also presented a variation of bacterial profile with IL-2 treatment (*P* = 0.001, **Supplementary Figure 2B**). The results of the comparative analysis showed that the Bacteroidaceae, Deferribacteraceae, and Prevotellaceae families were enriched, and the Ruminococcaceae family was depleted after IL-2 therapy (**Supplementary Figure 3A**). *Bacteroides*, *Mucispirillum*, *Lactococcus* and *Prevotella*_9 increased at the genus level, and [*Eubacterium*]_*coprostanoligenes*_*group Ruminiclostridium_6*, *Candidatus_Saccharimonas*, and Ruminococcaceae*_*UCD-014 decreased after IL-2 treatment (**Supplementary Figure 2C**).

Analysis of KEGG orthology suggested that the long-chain fatty acid transport protein (fadL), adenylyl-sulfate kinase (cysC), and fatty-acyl-CoA synthesis accumulated with IL-2 treatment (**Supplementary Figure 3B**). Meanwhile, enzymes involved in these KO contents were also revealed after IL-2 treatment (**Supplementary Figure 3C**). Signaling pathways such as steroidal hormone biosynthesis and mannan degradation have been enhanced by IL-2 treatment (**Supplementary Figures 2D**, **3D**).

To evaluate the immune system response to IL-2 treatment, we tested the lymphocytic profiles of the spleen and lymph nodes (including inguinal and cervical lymph nodes). The results indicated that Treg/Th17 and Treg/Tfh levels in the spleen and lymph nodes were significantly higher after IL-2 treatment (**Supplementary Figure 2E**). Interestingly, the enriched bacteria with IL-2 treatment were positively correlated with Treg/Th17 and Treg/Tfh rates and negatively associated with the proportions of the Th1, Th17, and the Tfh cells (**Supplementary Figure 2F**).

Together, these data indicate that administration of IL-2 could shape the bacterial community in mice with autoimmune disease and simultaneously suppress inflammation with the formation of plasticity of the host immune system. In a word, both the bacterial microbiome and immune response played a role in IL-2 therapy.

## Discussion

The IIMs are a heterogeneous group of chronic muscle disorders with therapeutic challenges. Till now, accumulating data suggest that both immune and nonimmune mechanisms cause muscle damage, and efficient treatment is mainly lacking (Zong and Lundberg, 2011). Immunological imbalance is considered a vital role in developing IIMs. Tregs are essential to maintaining the balance by suppressing the activation and expansion of auto-reactive T cells and other pathogenic immune cells (Hoeppli and Pesenacker, 2019). We and others have demonstrated the effectiveness of low-dose IL-2 therapy in IIMs, and associated with the expansion of Tregs and inhibition of Teff cells (Miao et al., 2021). As we know, IL-2 is the critical cytokine for the development and survival of Tregs, but other roles of IL-2 during treatment must be further explored.

Many studies found that gut microbiota dysbioses occur in different kinds of autoimmune diseases (De Luca and Shoenfeld, 2019), including IIMs. Certain microbial groups can be harmful and have other benefits for the balance of the autoimmune system. In our current study, the inflammatory-related bacteria, such as *Prevotella* increased, while some butyrate-producing bacteria, such as *Pseudobutyrivibrio, Lachnospiraceae, Roseburia*, and *Blautia*, decreased significantly. Several studies have proved that *Prevotellaceae* increased the risk for autoimmune diseases, such as RA, colitis, liver fibrosis, etc. (Garzoni et al., 2013; Jangi et al., 2016; Alpizar-Rodriguez et al., 2019). Studies in RA have proved that *Prevotella* may lead to loss of tolerance, which results in generating long-lived inflammatory effector T cells that drive chronic intestinal and extraintestinal inflammatory pathology in RA (Maeda et al., 2016; Alpizar-Rodriguez et al., 2019). Alternatively, many studies found that butyrate-producing bacterium can promote Tregs in the intestinal-related lymphatic system and are beneficial to restoring the immune conditions, leading to the decrease of risk of the pathogenesis of autoimmune diseases (O’Keefe, 2016; Chen et al., 2020).

The management of IIMs is challenging due to the heterogeneous nature of the disease and the lack of safe, effective, and specifically targeted therapies (Zong and Lundberg, 2011). We have shown that low-dose IL-2 can be used to treat IIMs, and was accompanied by a significant clinical improvement of IMACS DOI and notable improvements in clinical outcomes, including Fatigue, Heliotrope rash, Gottron’s sign, and CK level. In addition, low-dose IL-2 treatment led to a decrease in the steroid dose of active IIMs patients (Miao et al., 2021).

This study revealed that low-dose IL-2 treatment resulted in increased proportions of Tregs, which was consistent with previous clinical trials on IL-2 therapy in other autoimmune diseases. Besides restoring T cell homeostasis, IL-2 administration led to significantly altered gut microbiota and metabolite in serum and stool. Recently, it has become clear that microbiota and cellular metabolism elements are critical to immune cell development and differentiation, influencing both the development and effector functions of diverse immune cells (Hooper et al., 2012; Levy et al., 2017; Lin and Zhang, 2017). Low-dose IL-2 was observed to significantly alter the intestinal microbiota and metabolite in serum and stool, which is a novel mechanism for IL-2 in clinical use.

Severe gastrointestinal infection in mice leads to loss of T cell tolerance to commensal antigens and results in long-lived inflammatory effector T cells that drive chronic intestinal and extraintestinal inflammatory pathology (Hooper et al., 2012; Zilberman-Schapira et al., 2016; Lee, 2018). Because of the small sample size and lack of a control group, we next use NOD mice to analyze the gut microbiota’s alteration by low-dose IL-2 administration. In this study, some butyrate-producing bacteria, like *Bacteroidaceae* rosed considerably. Butyrate production is beneficial in improving growth and reducing the number of diseases, which plays a crucial role in maintaining the functional and morphology of intestinal epithelial cells and regulating the balance of intestinal flora.

Given the integrated nature of systemic metabolism, the analysis of multiple metabolites may better understand the disease-associated changes. Current studies have proved that intestinal symbiosis remarkably affected the metabolic and immune dysregulation in autoimmune diseases (Hooper et al., 2012; Levy et al., 2017; Lin and Zhang, 2017). We noted that low-dose IL-2 improved clinical responses of IIMs patients, together with dramatic alterations in metabolite profile. Interestingly, certain butyrate-producing bacteria were rosed remarkably after IL-2 treatment, associated with a rise in L-Asparagine and L-Leucine. L-asparagine has been used clinically as a significant combination of medications for treating hypertension, asthma, gastric cancer, etc. (Epp et al., 2016; Li et al., 2019), and has attracted considerable attention in the last few years due to remarkable anticancer properties (Li et al., 2019). L-Leucine is proven to join the mTOR pathway to increase Tregs and potentially treat autoimmune diseases. (Zeng et al., 2013; Shi et al., 2019). The microbiota-metabolism pathway altered by low-dose IL-2 needs to be further explored.

There are some limitations to this study. Firstly, reporting bias cannot be ruled out because limited sample size. There is a need to extend the sample size in the future to verify the conclusions. Second, the background treatment is different, and clinical stratification is lacking; thus future studies are needed. Third, in the animal study, because previous clinical studies had evaluated the safety of low-dose IL-2 in autoimmune conditions (Rosenzwajg et al., 2019; He et al., 2020), and some earlier studies have confirmed the efficiency of low dose IL-2 in mice (Grinberg-Bleyer et al., 2010; Ballesteros-Tato et al., 2012; He et al., 2016) similar to the dose used in our study, a toxicity study haven’t been carried out. Fourth, to support the findings of gut microbiota dysbiosis in human, the change in the gut microbiota before and after low dose IL-2 therapy in NOD mice was evaluated in supplementary material, though the results were weak. Further studies including control group are in need to overcome these weaknesses. Fifth, the online PICRUSt2 was performed to analyze the bacteria-associated signaling pathways. It allowed us to perform function prediction, but it predicted based on phylogenetic based functionality prediction. It is generally acknowledged that the PICRUSt2 is less effective than metabolomic and metatranscriptomic, so the results of function prediction needs further confirmation.

In summary, our study illustrates the gut microbiome composition and function in human and animal models with and without low-dose IL-2 treatment. Low-dose IL-2 induced the composition and taxa with specialized functions that have the potential to shape the stable states of the gut environment. Future studies will undoubtedly yield exciting new insights into how the IL-2 regulates immune-cell function and inflammation in IIMs through commensal microbiota. It will be important to understand the relationship between the treatment and gut microbiota and the composition and characteristics of the microbiome that affect autoimmune conditions and modulate susceptibility not only to IIMs but also to neurological, metabolic, and other autoimmune diseases. Further studies should inform new approaches for manipulating the microbiome to alter disease susceptibility and improve treatment efficacy.

## Data Availability Statement

The original contributions presented in this study are publicly available. This data can be found here: https://ngdc.cncb.ac.cn/gsa, CRA004579, and CRA004577.

## Ethics Statement

Ethics approval was obtained from the Peking University People’s Hospital Ethics Committee and was performed following the provisions of the Declaration of Helsinki and the International Council for Harmonisation guidelines for Good Clinical Practice. Written informed consent was obtained from all the enrolled patients or their legal representatives if they were unable to provide consent. The human ethics approval number is 2019PHB089. The animal ethics approval number is 2017PHC062.

## Author Contributions

YZ and JX conducted the data analysis and manuscript writing. MM completed the experiment and conducted manuscript writing. YML and YG helped in data analysis. YW, BH, and JT helped in animal experiment. JH conceived and designed the experiments. JH, YHL, JL, XS, DL, and ZL supervised the study and wrote the manuscript. All authors contributed to the article and approved the submitted version.

## Funding

This work was funded by the Beijing Sci-Tech Program (Z191100006619114), National Natural Science Foundation of China (U1903210, 82000496, 81801617, 82071813), Beijing Municipal Natural Science Foundation (7214267), Clinical Medicine Plus X-Young scholars Project of Peking University (PKU2021LCXQ025), and Peking University People’s Hospital Research and Development Foundation (RDX2020-03, RDY2020-21).

## Conflict of Interest

The authors declare that the research was conducted in the absence of any commercial or financial relationships that could be construed as a potential conflict of interest.

## Publisher’s Note

All claims expressed in this article are solely those of the authors and do not necessarily represent those of their affiliated organizations, or those of the publisher, the editors and the reviewers. Any product that may be evaluated in this article, or claim that may be made by its manufacturer, is not guaranteed or endorsed by the publisher.

## Supplementary Material

Supplementary Figure 1 PiCRUSt-based examination of the gut microbiome of the IIMs patients and the healthy controls. The online PICRUSt2 were performed to analyze the bacteria-associated signaling pathways. The signaling pathways were enriched depending on the MetaCyc database **(A)** and KEGG database **(B)**. Data visualization was performed using the R software (version 4.0.3) with the *ggplot2* package.

Supplementary Figure 2 The impact of low-dose IL-2 intervention on gut bacteria in NOD mice. **(A)** The α-diversity is measured by chao1, richness, Shannon_e, and equitability indexes. **(B)** The β-diversity is measured using the Adonis test by conditioned constrained principal coordinate analysis (CPCoA). **(C)** Significant differences of bacteria between the two groups at the genus level. **(D)** Signaling pathways enrichment analysis based on the MetaCyc pathway analysis. **(E)** Significant differences of immune parameters between the two groups. **(F)** Correlations between immune parameters and bacteria. Data visualization was performed using the R software (version 4.0.3) and the *corrplot* package **P* < 0.05; ***P* < 0.01; and ****P* < 0.001).

Supplementary Figure 3 Low-dose IL-2 affects the function of the bacteria in NOD mice. **(A)** Significant differences of bacteria between the two groups at the family level. The online PICRUSt2 was performed to analyze the bacteria-associated signaling pathways. The KEGG Orthology **(B)** and pathway **(D)** enrichment were analyzed depending on the KEGG database. Enzyme enrichment was analyzed depending on the MetaCyc database **(C)**. The differences of enzymes between the two groups. Data visualization was performed using the R software (version 4.0.3) with the *ggplot2* package.
