# Supplementary figures and images for: Modification of Intestinal Microbiota Dysbiosis by Low-Dose Interleukin-2 in Dermatomyositis: A Post Hoc Analysis From a Clinical Trial Study
Source: Front Cell Infect Microbiol. 2022 Mar 14;12:757099. doi: 10.3389/fcimb.2022.757099 (PMC8964112; doi:10.3389/fcimb.2022.757099)

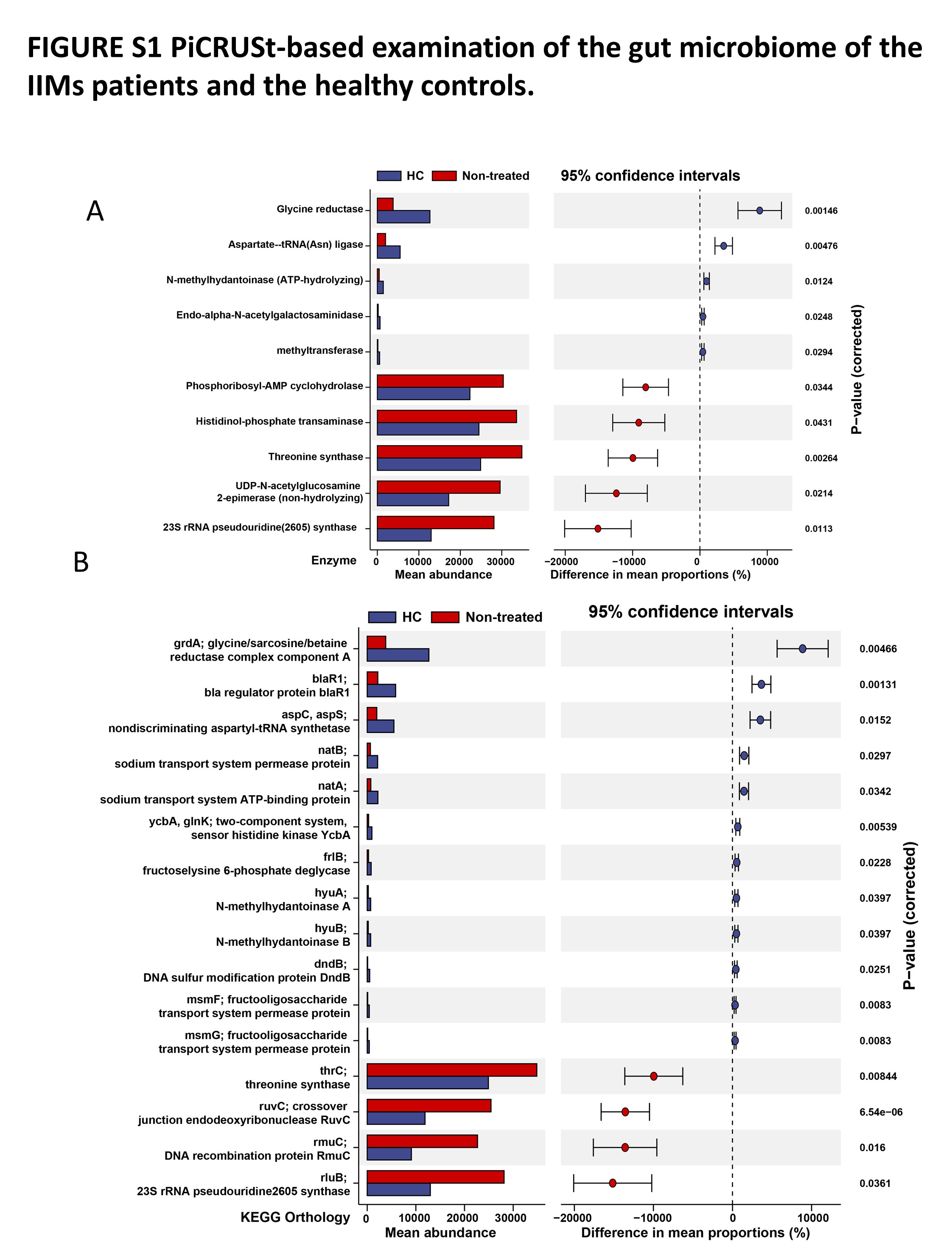

Supplement: Supplementary Figure 1 — PiCRUSt-based examination of the gut microbiome of the IIMs patients and the healthy controls. The online PICRUSt2 were performed to analyze the bacteria-associated signaling pathways. The signaling pathways were enriched depending on the MetaCyc database (A) and KEGG database (B). Data visualization was performed using the R software (version 4.0.3) with the ggplot2 package. [file Image_1.jpeg]

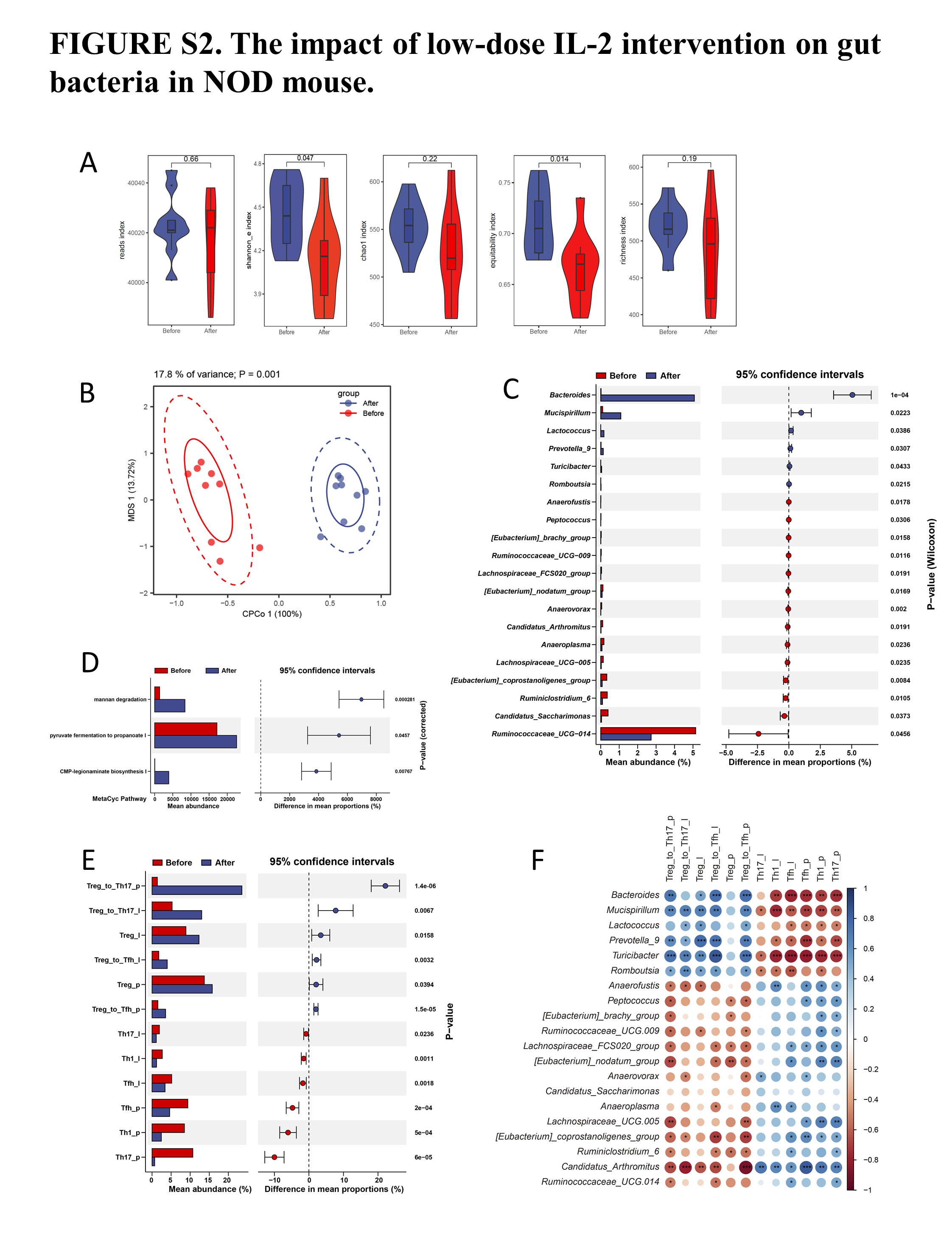

Supplement: Supplementary Figure 2 — The impact of low-dose IL-2 intervention on gut bacteria in NOD mice. (A) The α-diversity is measured by chao1, richness, Shannon_e, and equitability indexes. (B) The β-diversity is measured using the Adonis test by conditioned constrained principal coordinate analysis (CPCoA). (C) Significant differences of bacteria between the two groups at the genus level. (D) Signaling pathways enrichment analysis based on the MetaCyc pathway analysis. (E) Significant differences of immune parameters between the two groups. (F) Correlations between immune parameters and bacteria. Data visualization was performed using the R software (version 4.0.3) and the corrplot package *P < 0.05; **P < 0.01; and ***P < 0.001). [file Image_2.jpeg]

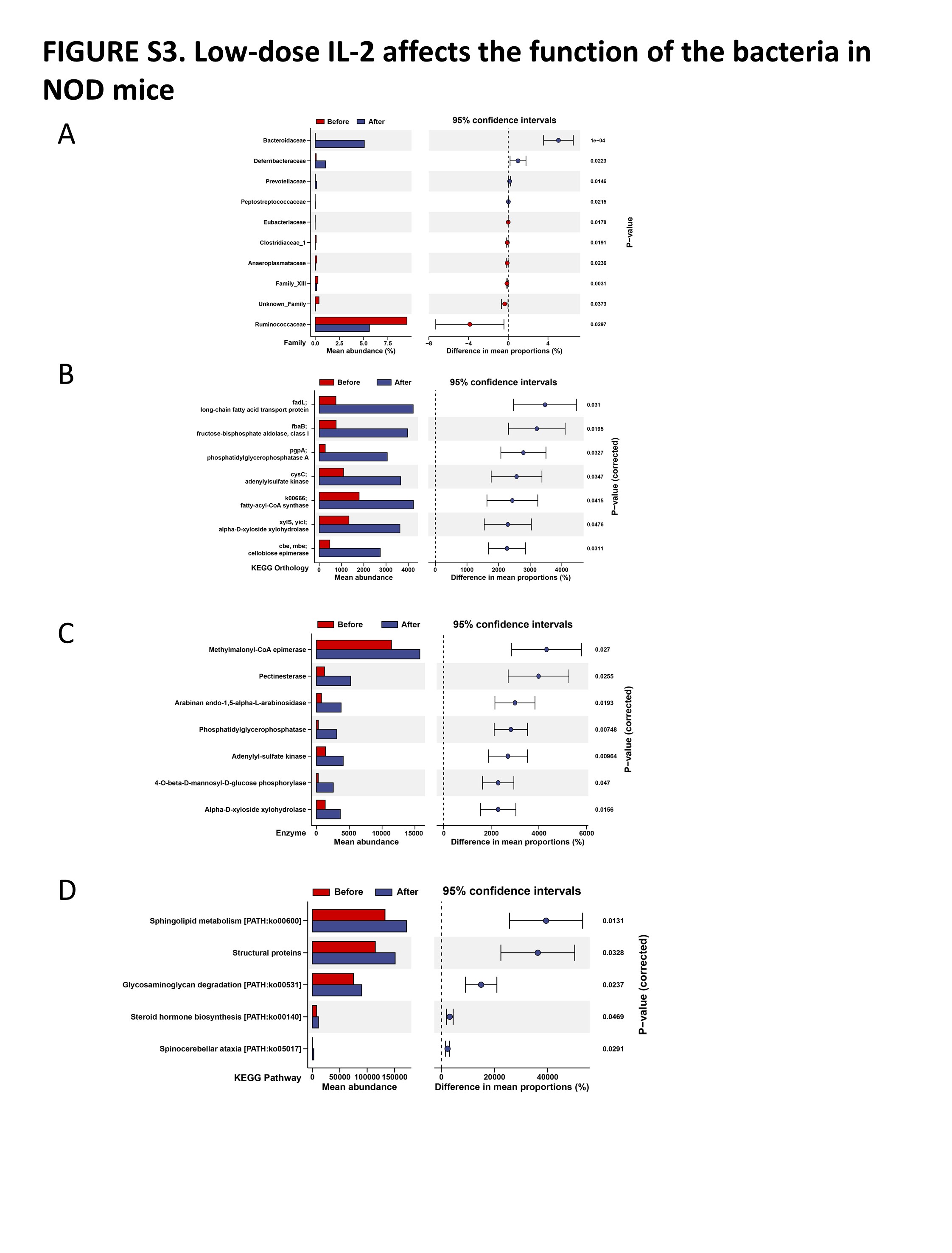

Supplement: Supplementary Figure 3 — Low-dose IL-2 affects the function of the bacteria in NOD mice. (A) Significant differences of bacteria between the two groups at the family level. The online PICRUSt2 was performed to analyze the bacteria-associated signaling pathways. The KEGG Orthology (B) and pathway (D) enrichment were analyzed depending on the KEGG database. Enzyme enrichment was analyzed depending on the MetaCyc database (C). The differences of enzymes between the two groups. Data visualization was performed using the R software (version 4.0.3) with the ggplot2 package. [file Image_3.jpeg]
