# Supplementary material for: Modification of Intestinal Microbiota Dysbiosis by Low-Dose Interleukin-2 in Dermatomyositis: A Post Hoc Analysis From a Clinical Trial Study
Source: Front Cell Infect Microbiol. 2022 Mar 14;12:757099. doi: 10.3389/fcimb.2022.757099 (PMC8964112; doi:10.3389/fcimb.2022.757099)
Supplement: Supplementary file 4 [file Table_1.docx]

**Supplementary Table 1: Immune index differentiation with IL-2 treatment**

| **Immune.index** | **conf.low** | **conf.high** | **t.test.p.value** | **wilcoxon.p.value** |
| --- | --- | --- | --- | --- |
| ESR | -9.897684492 | 16.51306911 | 0.608923595 | 0.97952616 |
| IgA | -1.62987927 | 1.672956193 | 0.978741467 | 1 |
| IgG | -2.894235806 | 4.525005037 | 0.653763143 | 0.448300867 |
| IgM | -0.896901398 | 1.008901398 | 0.904139567 | 0.79753247 |
| Cr | -7.866199598 | 8.943122675 | 0.895814775 | 0.959079799 |
| ALT | -12.01673502 | 21.55519655 | 0.562908953 | 0.425896013 |
| AST | -4.436383531 | 14.74407584 | 0.27638031 | 0.237481195 |
| WBC | -8.609105134 | 1.813720519 | 0.190990271 | **0.033827664** |
| Hb | -15.72456197 | 10.9553312 | 0.715244165 | 0.700424911 |
| PLT | -131.7128393 | 31.25130081 | 0.214947756 | 0.264211103 |
| TNF_alpha_B | -13.50248683 | 6.164025292 | 0.447358314 | 0.686631733 |
| IFN_gamma_B | -13.93582132 | 4.751205935 | 0.315342316 | 0.538231279 |
| IL_2_B | -4.978452516 | 21.33229867 | 0.211765227 | 0.389689633 |
| IL_4_B | -0.829921825 | 0.382229518 | 0.451117152 | 0.626013062 |
| IL_17_B | -0.579559409 | 0.247251717 | 0.414646125 | 0.504620634 |
| TNF_alpha_S | -101.9292356 | 77.93892795 | 0.785108066 | 0.959817703 |
| IFN_gamma_S | -59.77013604 | 28.1536745 | 0.462496944 | 0.840250947 |
| IL_2_S | -101.9511486 | 186.1217886 | 0.550945733 | 0.286857874 |
| IL_4_S | -5.004669126 | 6.068438357 | 0.843284316 | 0.418412976 |
| IL_17_S | -6.146679804 | 6.662295189 | 0.934171816 | 0.310733035 |
| CD127Treg_B | -7.836728326 | 1.405959096 | 0.16355383 | 0.130259547 |
| CD161Treg_B | -3.439858707 | 4.516781784 | 0.781919167 | 0.959086793 |
| CLATreg_B | -9.169178333 | 3.584562949 | 0.37414044 | 0.572553031 |
| pTfh_B | -0.465908259 | 1.332574926 | 0.328301863 | 0.304647122 |
| Native.Th_B | -22.68799819 | 8.785434091 | 0.370846048 | 0.335833894 |
| Foxp3_B | -9.129459701 | -0.304386453 | **0.037415724** | 0.112932523 |
| Teff_B | -0.070734642 | 9.045093616 | **0.053319382** | 0.12380728 |
| Th17_to_Foxp3Treg | -0.017340973 | 0.119935071 | 0.134142463 | 0.286857874 |
| Teff_to_Treg | -0.837589714 | 7.443884528 | 0.112708034 | 0.112932523 |
| Treg_to_Th17 | -4.301410581 | 1.385937976 | 0.300586465 | 0.286857874 |
| Treg_to_Teff | -0.126109851 | -0.004746652 | **0.036038797** | 0.112932523 |
| CD127Treg_S | -47.52826363 | 44.03503287 | 0.93787951 | 0.613869777 |
| CD161Treg_S | -25.02879939 | 53.26141478 | 0.463911264 | 0.418412976 |
| CLATreg_S | -42.22605049 | 97.62451203 | 0.417186176 | 0.801041863 |
| pTfh_S | -7.712013488 | 13.3997058 | 0.583319491 | 0.166097687 |
| Native.CD4+T_S | -165.5531747 | 262.5233286 | 0.642436541 | 0.919734438 |
| Foxp3_S | -64.72827855 | 35.73390932 | 0.554362678 | 0.801041863 |
| Teff_S | -162.240883 | 415.7796522 | 0.374233206 | 0.36215776 |
| T_B | -2.157971893 | 18.08104882 | 0.117375314 | 0.168978136 |
| CD4+T_B | -2.071336211 | 20.28672083 | 0.105637881 | 0.208889428 |
| CD8+T_B | -10.73394596 | 8.05702288 | 0.771138849 | 0.938663807 |
| T_S | -458.2660514 | 694.5737437 | 0.676006652 | 0.54460435 |
| CD4+T_S | -280.4821466 | 395.2513774 | 0.728930379 | 0.613869777 |
| CD8+T_S | -88.80532062 | 191.2668591 | 0.456808398 | 0.686631733 |
| CD4_to_CD8 | -0.179822964 | 1.101361425 | 0.149847716 | 0.199670876 |
| B_B | -11.72892501 | 6.575078852 | 0.566491542 | 0.758196306 |
| B_S | -482.6825695 | 553.144108 | 0.88951455 | 0.762310828 |
| NK_B | -345.1384736 | 362.1384736 | 0.960834183 | 0.335833894 |
| NK_S | -357.8507386 | 107.8507386 | 0.27664272 | 0.054429114 |
| LY_S | -688.660173 | 822.1986346 | 0.856386941 | 0.762310828 |

ESR, erythrocyte sedimentation rate. IgA, immunoglobulin A. IgG, immunoglobulin G. IgM, immunoglobulin M. Cr, creatinine. ALT, alanine transaminase. AST, aspartate transaminase. WBC, white blood cell. Hb, hemoglobin. PLT, platelet. TNF_alpha, tumor necrosis factor-α. IFN_gamma, interferon-γ. IL-2, interleukin-2. IL-4, interleukin-4. IL-17, interleukin-17. Treg, regulatory T cell. CLATreg, cutaneous lymphocyte-associated Ag regulatory T cell. pTfh, peripheral T follicular helper cells. Native.Th, native T helper cell. Teff, effector T cell. NK, natural killer cell.

**Supplementary Table 2: Metabolic index differentiation with IL-2 treatment**

| serum | conf.low | conf.high | t.test.p.value | wilcoxon.p.value |
| --- | --- | --- | --- | --- |
| L-3-Amino isobutyric acid | -32.7069 | 115.3169 | 0.25946124 | 0.347357919 |
| D-alloThreonine | -355.671 | 120.6045 | 0.310952711 | 0.629725504 |
| L-2-Aminobutyric acid | -205.993 | 452.5175 | 0.444144082 | 0.551167166 |
| O-Acetyl-L-serine | -433.22 | 277.8319 | 0.651514844 | 0.347357919 |
| L-Glutamine | -1121.38 | 440.603 | 0.375733426 | 0.347357919 |
| L-Kynurenine | -69.4401 | 166.5964 | 0.401851088 | 0.377685311 |
| L-Asparagine | -391.904 | -6.19442 | **0.043658127** | 0.059657061 |
| Pipecolic acid | -4138.96 | -65.1591 | **0.04382681** | **0.044901995** |
| L-Methionine | -404.741 | 636.6889 | 0.64597498 | 0.44283318 |
| L-Proline | -653.271 | 383.1782 | 0.594319178 | 0.551167166 |
| r-AminobutyrIec Acid | 3.668211 | 30.89766 | **0.016123855** | **0.006811737** |
| L-Isoleucine | -254.211 | 193.9005 | 0.781922316 | 0.078024345 |
| L-Tyrosine | -438.121 | 150.3353 | 0.318192139 | 0.409510398 |
| L-Histidine | -952.404 | 32.28571 | 0.065485078 | 0.1276879 |
| L-Arginine | -2137.5 | 182.7663 | 0.094471945 | 0.088733786 |
| L-Glutamic acid | -475.147 | 263.3199 | 0.554737435 | 0.377685311 |
| Glycine | -12984 | 2246.374 | 0.156113477 | 0.159973019 |
| L-Phenylalanine | -342.128 | 206.6797 | 0.613197157 | 0.755284828 |
| Sarcosine | -2548.97 | 314.9702 | 0.119968004 | 0.059657061 |
| L-Tryptophan | -514.772 | 113.4797 | 0.19885928 | 0.218920802 |
| L-Citrulline | -520.036 | 94.76421 | 0.165038816 | 0.197808115 |
| L-Leucine | -355.744 | -65.4019 | **0.006935252** | **0.003636625** |
| L-Alanine | -1548.69 | 21.74245 | 0.056003137 | 0.088733786 |
| L-Serine | -991.297 | 428.3394 | 0.414333707 | 0.887385935 |
| beta-Alanine | -375.52 | 47.9839 | 0.122785795 | 0.241523788 |
| Hypotaurine | -63.8951 | 6.185556 | 0.101548617 | 0.143167776 |

| **fecal** | **conf.low** | **conf.high** | **t.test.p.value** | **Wilcoxon.p.value** |
| --- | --- | --- | --- | --- |
| L-3-Amino isobutyric acid | -9.475999598 | 3.211532931 | 0.306605576 | 0.755284828 |
| D-alloThreonine | -24.31071787 | 6.845967868 | 0.254863899 | 0.113502328 |
| L-2-Aminobutyric acid | -75.38273949 | 15.17298949 | 0.174931221 | 0.551167166 |
| O-Acetyl-L-serine | -177.0948854 | 64.64246869 | 0.331407469 | 0.409510398 |
| L-Glutamine | -10.94188996 | 0.751056631 | 0.082069063 | 0.068361441 |
| Pipecolic acid | -33.48056243 | 9.327829097 | 0.24351806 | 0.84283599 |
| L-Methionine | -283.0315507 | 45.41150074 | 0.142992993 | 0.347357919 |
| L-Proline | -15.20511171 | 6.402761707 | 0.406990774 | 0.377685311 |
| r-AminobutyrIec Acid | -21.12070187 | 23.5017852 | 0.912587804 | 0.712535076 |
| L-Isoleucine | -56.09676013 | 10.22129347 | 0.159326375 | 0.347357919 |
| L-Tyrosine | -96.393666 | 6.832716003 | 0.084739746 | 0.1276879 |
| L-Histidine | -40.02590131 | 11.47403465 | 0.254209888 | 0.1276879 |
| L-Arginine | -9.496561021 | 13.43071102 | 0.722932926 | 0.932300503 |
| L-Glutamic acid | -184.2680243 | 69.14744099 | 0.342568062 | 0.377685311 |
| Glycine | -902.3266372 | 736.4717205 | 0.83382738 | 0.178182028 |
| L-Phenylalanine | -50.87588712 | 5.083303783 | 0.102169159 | 0.241523788 |
| Sarcosine | -426.7465022 | 193.8461522 | 0.439784473 | 0.197808115 |
| L-Tryptophan | -18.37709027 | 4.934256941 | 0.244443652 | 0.113502328 |
| L-Citrulline | -108.5199781 | 15.12942808 | 0.128105323 | 0.159973019 |
| L-Leucine | -95.49257071 | 22.26698738 | 0.204752949 | 0.44283318 |
| L-Alanine | -255.8708618 | 171.6762785 | 0.686882966 | 0.178182028 |
| L-Serine | -49.2639453 | 17.7943953 | 0.336541397 | 0.31858517 |
| beta-Alanine | -66.97100402 | 53.51310402 | 0.818950407 | 0.291342659 |
